# Reactive silica nanoparticles turn epoxy coating from hydrophilic to super-robust superhydrophobic†

**DOI:** 10.1039/c8ra10046b

**Published:** 2019-04-24

**Authors:** Danfeng Zhi, Huanhuan Wang, Dong Jiang, Ivan P. Parkin, Xia Zhang

**Affiliations:** Engineering Research Center for Nanomaterials, Henan University Kaifeng 475004 PR China xia.zhang@ucl.ac.uk; State Key Laboratory of Solid Lubrication, Lanzhou Institute of Chemical Physics, Chinese Academy of Sciences Lanzhou 730000 PR China; Department of Chemistry, University College London 20 Gordon Street London WC1H 0AJ UK

## Abstract

Superhydrophobic organic–inorganic hybrid nanocomposite coatings have received much attention because they possess the advantages of both inorganic and organic materials. Nevertheless, it is difficult to achieve strong bonding of inorganic nanoparticles to the polymer matrix while maintaining a sufficiently rough structure to impart superhydrophobicity. In this study, we fabricated silica nanoparticles with surface reactive groups that can further react with the epoxy resin. Thus, the hydrophobic silica nanoparticles were stably anchored and stabilized in the cured resin matrix while forming nanometer-scale roughness structures. The obtained silica-decorated epoxy resin coating shows great durability and water-repellency after mechanical abrasions and has superior adhesion to the substrate.

## Abstract 2

Superhydrophobic organic–inorganic hybrid nanocomposite coatings have received much attention because they possess the advantages of both inorganic and organic materials.

## Graphical abstract

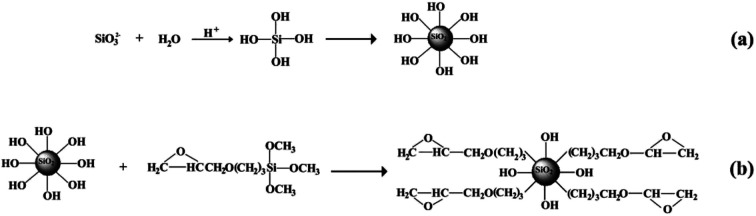

## 1. Introduction

Coating is an essential step in adjusting the surface properties of materials.^1^ Superhydrophobic coatings have received much attention because of their applications in fields such as self-cleaning,^2–4^ non-wetting liquids,^5^ anti-icing^6–8^ and oil–water separation.^7,8^ A superhydrophobic coating surface is usually constituted by a combination of low surface energy substances and micro/nanometer scale roughness structures.^9–11^ The latter, however, always have poor mechanical strength and weak adhesion to the substrates, seriously hindering the large-scale preparation and industrial application of superhydrophobic surfaces.^12–14^ Introducing nanomaterials into polymers to increase the surface nano-scale roughness has been demonstrated as an excellent strategy for producing low-cost, durable, superhydrophobic nanocomposite coatings.^15–18^ However, the inorganic nanoparticles generally have poor compatibility with organic polymers or solutions, which leads to phase separation during fabrication and tends to diminish the quality of the coating through cracks or weak adhesion to the substrates.^19,20^ To overcome this problem, Barron *et al.* prepared superhydrophobic surfaces by combining the surface roughness of nanoparticle-derived films with the low surface energy properties of highly branched alkyl chains.^21^ Bayer *et al.* fabricated wear- and abrasion-resistant high-impact polystyrene/silica nanocomposite coatings for metal surfaces by a spray method.^22,23^ In our previous work, we have also fabricated superhydrophobic epoxy resin coatings by adding hydrophobic SiO_2_ nanoparticles modified by hexamethyldisilazane.^24^ Though surface modification can improve the compatibility between the inorganic nanoparticles and organic material to a certain extent, nanoparticles typically still need strong bonds with polymers to construct a structure with durable roughness. If the nanoparticles are just slightly adhered to or sputtered on the layer surface, roughness can be obtained but it is easily damaged or removed under mechanical abrasion.^25^ At present, an important approach is to introduce functional groups on the nanoparticle surfaces and endow the nanoparticles with reactive properties, which enable the nanoparticles to take part in chemical reactions and anchor on to the polymer matrix.^26^

In past researches, epoxy resin has been widely used to strengthen the adhesion of nanoparticles, especially silica nanoparticles, which are easy to prepare, have synthetically-controlled diameters, and possess a high concentration of Si–OH groups suitable for modification. These have been widely introduced into the resin matrix as hydrophobic fillers to fabricate superhydrophobic surfaces.^27–30^ Ramakrishna *et al.* reported the preparation of a superhydrophobic coating using oligomer-wrapped silica nanoparticles obtained through reactions between silanol and isocyanate groups.^31^

In this study, we first fabricated surface functional silica nanoparticles and used them as a SiO_2_/epoxy resin solution that was sprayed on hard or soft substrates to fabricate superhydrophobic surfaces. By introducing surface functional silica nanoparticles, the inherently hydrophilic epoxy resin coating can be turned into a superhydrophobic one with mechanical abrasion resistance. There are two advantages in this method: (1) by virtue of the interaction between the epoxy groups on the functionalized silica nanoparticle surface and the primary amine group of the curing agent, silica nanoparticles were stably anchored onto the cured epoxy resin matrix while constructing micrometer-scale roughness structures on the coating surface; (2) epoxy resin is an adhesive itself and using epoxy resin as a coating material can resolve the problem of poor adhesion between the coating and substrates.

## 2. Experimental section

### 2.1 Preparation of superhydrophobic silica powder

**Fig. 1:**
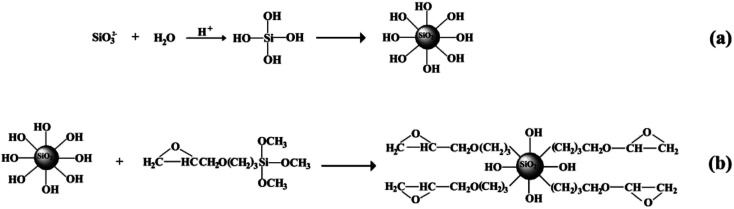
Schematic illustration of KH560 functionalization of silica nanoparticles.

The surface functionalized silica nanoparticles were prepared by an *in situ* surface modification method and Fig. 1 schematically shows the preparation processes. Briefly, sodium metasilicate and hydrochloric acid were dissolved in deionized water, separately, and the γ-(2,3-epoxypropoxy)propyltrimethoxysilane (KH560) was dissolved in absolute alcohol as a modifier. Then the solution of hydrochloric acid and KH560 were added into the sodium metasilicate solution dropwise. Gradually, an amount of foam appeared on the surface of this aqueous solution and the suspension was heated to 60 °C and was stirred for 4 h at this temperature. The suspension separated into two layers quickly, and a layer of white floc floated on the top. The floc was collected by filtration and washed clearly. Finally, the filtered cake was re-dispersed into a quantity of mixed solution to form an emulsion, and the emulsion was spray-dried at 140 °C to get the white SiO_2_ nanoparticles. A typical formula was used as follows: 0.3 mol L^−1^ sodium metasilicate, 0.72 mol L^−1^ hydrochloric acid, and 0.05 mol L^−1^ modifier. The detailed information about surface functionalized SiO_2_ nanoparticles are shown in S1 in the ESI.†

### 2.2 Preparation of silica/epoxy resin superhydrophobic coatings

Briefly, 10 g of epoxy resin, 3 g of the triethylenetetramine as curing agent and 4 g of hydrophobic silica nanoparticles were dispersed in 150 g of xylene to make a uniform suspension. Then, the obtained suspension was spin-coated (2500 rpm, 20 s) on hard or soft substrates and cured at 140 °C for 1.5 h to get the silica/epoxy resin superhydrophobic coatings.

### 2.3 Characterization

The surface morphology of the samples was examined with a field emission scanning electron microscope (FESEM, JSM-6701F). The surface components of the samples were analyzed with an X-ray photoelectron spectrometer (XPS, AXIS ULTRA). Thermogravimetric (TG) measurements were performed with a NETZSCH STA 449 C at a dynamic heating rate of 10 °C min^−1^. The contact angles (CAs) and sliding angles (SA) of water were measured using a DSA-100 optical contact angle meter (Kruss Company, Ltd, Germany) at room temperature (22 °C). The average CA value was determined by measuring the same sample at five different positions. The volume of all liquids was 5–8 μL when the contact angles and sliding angles were measured. The image of the droplet was obtained with a digital camera (NIKON, P600). The transparency of the coatings was tested using a UV-vis spectrometer (UV-1800PCS, Mapada Instruments). The transmittance of each sample was recorded at 340–800 nm and collected at an ambient temperature.

### 2.4 Abrasion test

Abrasion tests were carried out on the SiO_2_/epoxy resin coating as well as the purchased commercial Never Wet coating. In this test, the coating was placed face-down on the sandpaper (standard glass paper, Grit No. 800) and moved 23 cm, then rotated by 90° (face to the sandpaper) and moved 20 cm. This process is defined as one abrasion cycle, which guarantees the surface abrasion longitudinally and transversely in each cycle, even if it is moved in a single direction.

## 3. Results and discussion

### 3.1 Superhydrophobic silica-decorated nanocomposite coating

**Fig. 2:**
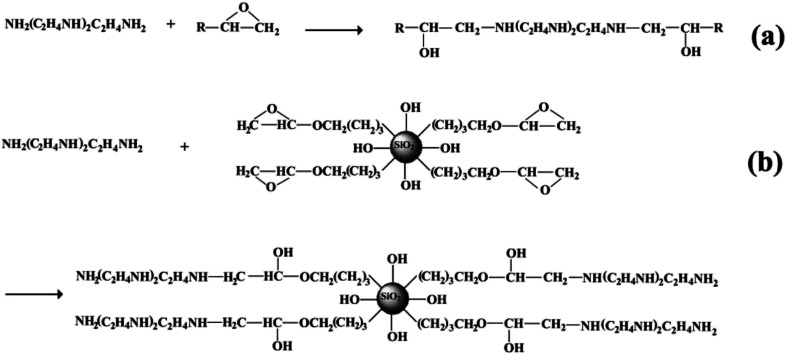
Schematic illustration of surface functionalized silica nanoparticles cured with epoxy resin.

**Fig. 3:**
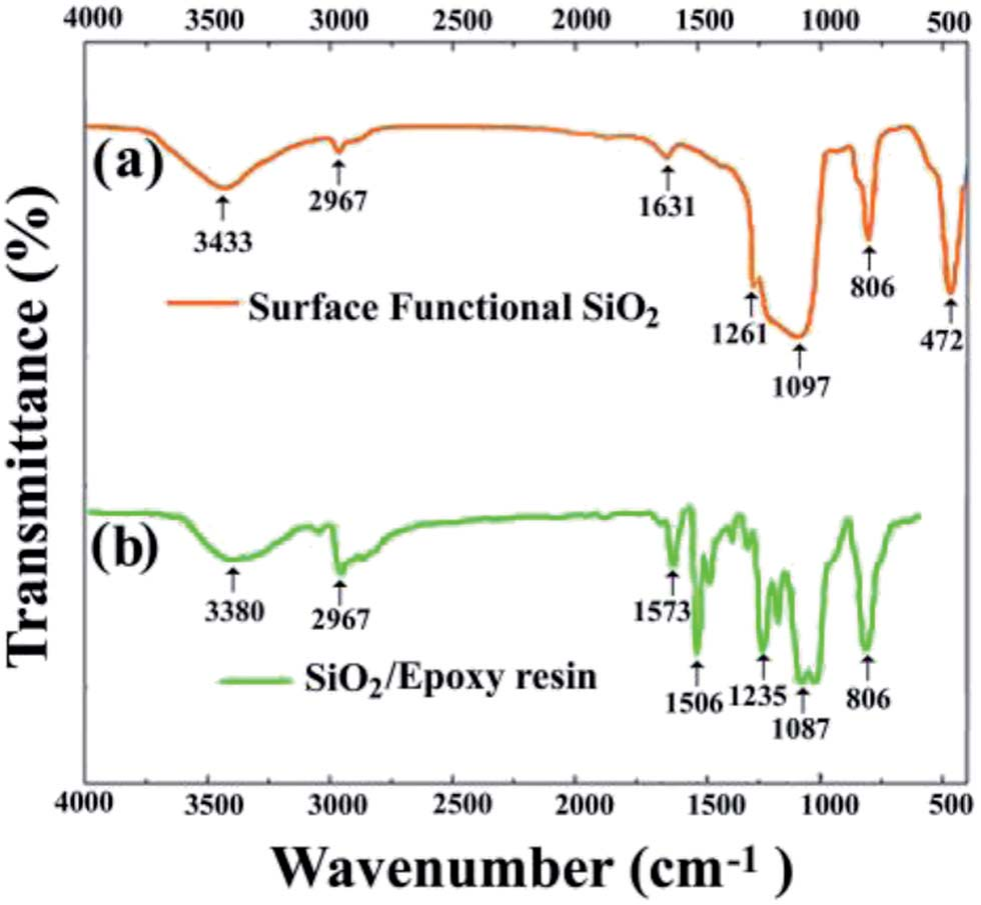
(a) FTIR of surface functional SiO_2_ nanoparticles and (b) SiO_2_/epoxy resin coatings.

The reactive surface-functionalized SiO_2_ nanoparticles can take part in the curing process along with the epoxy resin and are anchored to the coating. The reaction process is shown in Fig. 2. Fig. 3 shows the infrared spectrum of the obtained surface-functionalized SiO_2_ nanoparticles and the SiO_2_/epoxy resin composite coating. As can be seen from Fig. 3(a), the peaks at 1087 cm^−1^, 806 cm^−1^ and 472 cm^−1^ are characteristic peaks for SiO_2_. The weak peak at 1261 cm^−1^ is attributed to the epoxy group's stretching vibration, which indicates that the epoxy groups have successfully been modified on the SiO_2_ nanoparticle surface.^32–34^Fig. 3(b) shows the obtained SiO_2_/epoxy resin composite coating after completing the curing process, in which an epoxide-ring opening occurs between the epoxy ring of the epoxy resin and the primary amine group of the triethylenetetramine. This can be confirmed from the diminished intensity of the characteristic bands of the epoxide ring (*i.e.*, 915 cm^−1^ and 831 cm^−1^) and primary amine (*i.e.*, 3206/3316 cm^−1^), as well as the increased intensity of the –OH band (*i.e.*, 3380 cm^−1^) and secondary amine groups (*i.e.*, 1573 cm^−1^).^35–37^

**Fig. 4:**
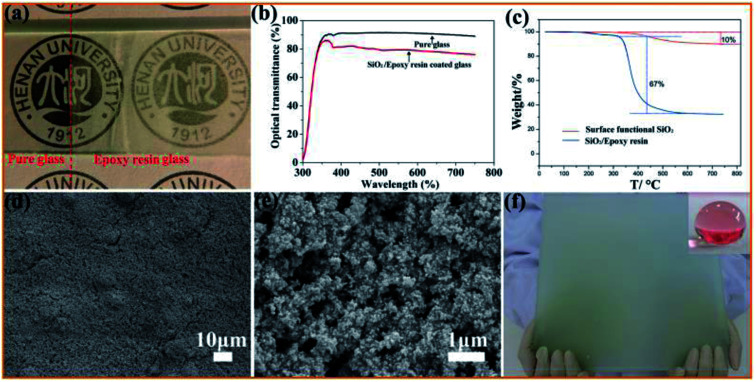
(a) Photograph of the SiO_2_/epoxy resin coating on glass; (b) transmittance spectrum of the glass slide before and after coating with SiO_2_/epoxy resin. (c) Thermogravimetric analysis of the surface functional nanoparticles and SiO_2_/epoxy resin coatings; (d and e) SEM images of the coating surface. (f) Large size (45 × 35 cm) of the as-prepared SiO_2_/epoxy resin coating with superhydrophobicity.

Fig. 4(a) shows the optical images of glass before and after being coated with a SiO_2_/epoxy resin coating with a thickness of about 7 μm, and all the letters show good readability under the coated glass. The translucent coatings demonstrate excellent superhydrophobic properties and water droplets dropped on the coating maintain nearly spherical shapes with a contact angle of 156° and sliding angle of about 3°. The transmittance spectrum of the coated glass in the visible region (300–800 nm) is a rational characterization of transparency, as shown in Fig. 4(b).

In order to investigate the thermal stability of the coating, thermogravimetry (TG) was performed, as shown in Fig. 4(c). It is clear that almost no weight loss occurs before 200 °C, and between 200 °C and 500 °C, the SiO_2_/epoxy resin begins to decompose and the weight loss reaches 67%. As far as the surface functionalized SiO_2_ nanoparticles are concerned, there is a weight loss of about 10% between 200 °C and 380 °C, which is due to thermal decomposition of the surface functional groups. To further investigate the thermal coating stability, we also analyzed how the contact angle and sliding angle changed with the curing temperature. It was found that when the curing temperature was higher than 320 °C, the superhydrophobicity was destroyed and the sliding contact angle increases sharply (Fig. S2†), which further indicates that the surface functional reagents that are necessary for superhydrophobicity undergo thermal decomposition. From the SEM observation, it can be seen that SiO_2_ nanoparticles have been constructed with micrometer-scale roughness structures with lots of cavities on the surface. In addition, the coating can be easily fabricated on a large scale by a spray method as shown in Fig. 4(f), which is very important for SiO_2_/epoxy resin coatings in a wide range of applications in the real world environment. Note that the coating (Fig. 4(f)) becomes non-transparent because the transmittance of the coating decreases with the increase in the coating thickness. Detailed information is shown in Fig. S3,† where it was found that when the coating thickness is 30.37 μm, the optical transmittance is only 20%, indicating that the coated glass can turn almost opaque.

Herein, the anchoring of SiO_2_ nanoparticles plays an important role in adjusting the coating wetting behavior. Through surface modification, hydrophilic SiO_2_ converts to hydrophobic SiO_2_, and the aggregations of SiO_2_ nanoparticles affect the coating surface roughness as well as reduce the surface energy that are responsible for inducing the superhydrophobic behavior. To investigate the influence of SiO_2_ nanoparticle content on the wettability of the coating, different SiO_2_ nanoparticles were added, as shown in Fig. S4.† The coating wettability gradually switches from hydrophilic to hydrophobic with an increase in the SiO_2_ content. From the SEM images, it is clear that the coating has a rough surface and the surface roughness increases with an increase in the content of SiO_2_ nanoparticles (Fig. S5†), which is very important for producing superhydrophobicity.

### 3.2 Mechanical durability

**Fig. 5:**
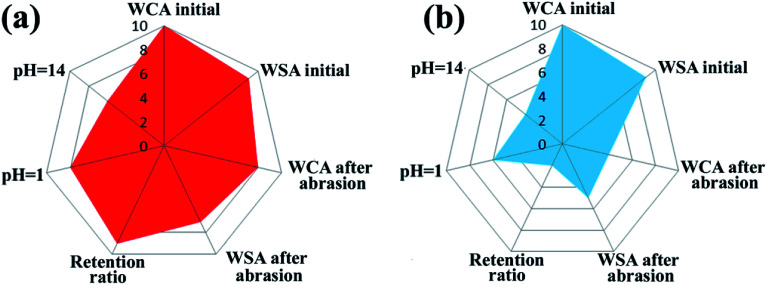
Radar diagrams of SiO_2_/epoxy coatings (a), and the “Never Wet” coatings (b) a commercial superhydrophobic paint. Here, “WCA initial” and “WSA initial” refer to the water contact angles and water sliding angles of the samples without any mechanical and chemical tests. “WCA after abrasion” and “WSA after abrasion” refer to the water contact angles and water sliding angles that were measured after the samples being abraded for 4000 cm (2 kPa pressure, SiC, 800 Cw sandpaper). “pH = 1” and “pH = 14” refer to the contact angles that were measured after 60 min “droplet test”.

To give an overview of the mechanical and chemical durability of the SiO_2_/epoxy coating, and to compare it with commercial superhydrophobic surfaces, we used radar diagrams to evaluate the experimental data as shown in Fig. 5. In the radar diagrams, we included water contact angles and sliding angles before and after sandpaper abrasion (SiC, 8000 Cw, 2 kPa pressure, 4000 cm abrasion, and the thickness of both the obtained coating and commercial coating was 75–80 μm), retention ratio after 4000 cm abrasion, and contact angle after the “droplet tests” for 60 min; Table 1 shows the rating system of the radar diagram according to the performance of the samples, and their data sheets are shown in Table 2. The larger overall area in the plot indicates that the material can perform better. Compared with the commercial superhydrophobic coating (Never Wet), the obtained SiO_2_/epoxy resin coating shows superior chemical and mechanical abrasion resistance.

**Table 1:** Classification system for radar diagram

Radar diagram point	WCA, *θ* [deg]	WSA, *α* [deg]	Retention ratio, *η* [%]
1	*θ* ≤ 120	*α* > 33	*η* ≤ 85
2	120 < *θ* ≤ 125	29 < *α* ≤ 33	85 < *η* ≤ 86
3	125 < *θ* ≤ 130	25 < *α* ≤ 29	86 < *η* ≤ 87
4	130 < *θ* ≤ 135	21 < *α* ≤ 25	87 < *η* ≤ 88
5	135 < *θ* ≤ 140	17 < *α* ≤ 21	88 < *η* ≤ 89
6	140 < *θ* ≤ 145	13 < *α* ≤ 17	89 < *η* ≤ 90
7	145 < *θ* ≤ 150	9 < *α* ≤ 13	90 < *η* ≤ 91
8	150 < *θ* ≤ 155	5 < *α* ≤ 9	91 < *η* ≤ 92
9	155 < *θ* ≤ 160	1 < *α* ≤ 5	92 < *η* ≤ 93
10	*θ* > 160	*α* ≤ 1	*η* > 93

**Table 2:** Average values and errors of performance characteristics and their corresponding points on the radar diagram

Priority	SiO_2_/epoxy resin	Never Wet
WCA initial [deg]	161.5 ± 2.5 (10)	160 ± 1.8 (10)
WSA initial [deg]	3.5 ± 1.2 (9)	3.8 ± 1.0 (9)
WCA after abrasion [deg]	151.0 ± 2.1 (8)	130 ± 3.7 (4)
WSA after abrasion [deg]	11.5 ± 1.3 (7)	19.8 ± 1.4 (5)
Retention ratio [%]	92.5 ± 0.4 (9)	85.2 ± 0.7 (2)
WCA pH = 1 [deg]	153.0 ± 2.0 (8)	143.5 ± 1.5 (6)
WCA pH = 14 [deg]	140.5 ± 2.3 (6)	132.5 ± 2.2 (4)

**Fig. 6:**
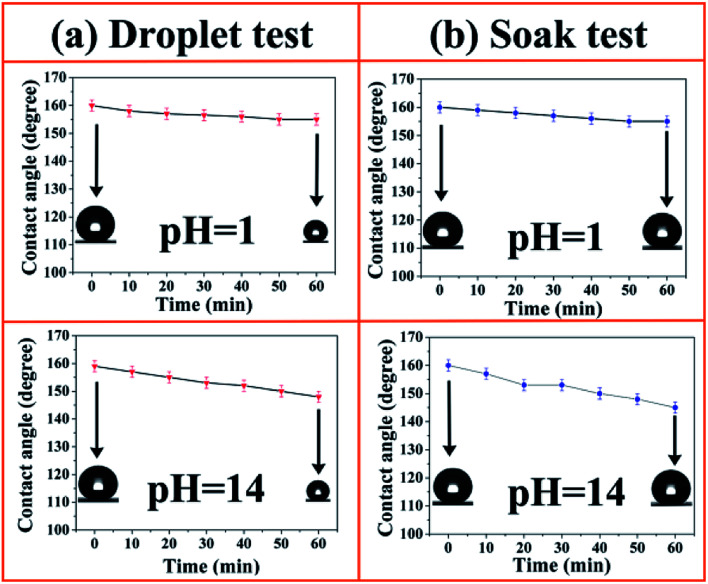
Contact angle as a function of time in acidic/alkali droplet contact tests (a) and acidic/alkali soak tests (b) on SiO_2_/epoxy coatings.

The chemical durability of the SiO_2_/epoxy resin coatings was studied by two independent experiments and the detailed information is shown in Fig. 6. In the “droplet test”, strong acid (pH = 1) and alkali (pH = 14) droplets were placed on the SiO_2_/epoxy coating for 60 min as shown in Fig. 6(a). The water droplets became smaller as time passed because of evaporation. As the acid/alkali contact time increased, the CAs of acid/alkali droplets slightly decreased but still remained about 150°. In a more aggressive test, the samples were immersed into acid (pH = 1) and alkali (pH = 14) baths for 60 min, and CAs were measured for every 10 min of soak time as shown in Fig. 6(b). Although the WCAs decreased from 160° to about 150°, the SiO_2_/epoxy coatings retained their superhydrophobicity.

**Fig. 7:**
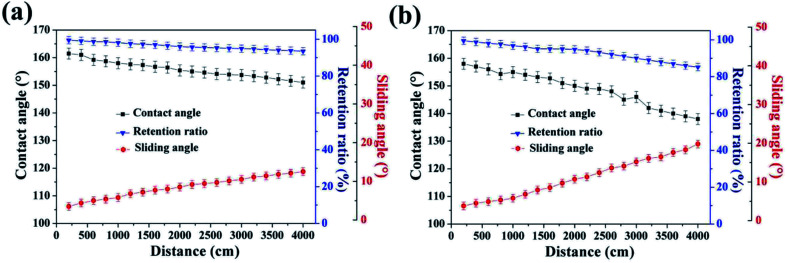
Plot of water contact angle, sliding angle and retention ratio as a function of sandpaper abrasion distance (2 kPa pressure, SiC, 800 Cw sandpaper) on SiO_2_/epoxy coatings (a) and Never Wet (b).

**Fig. 8:**
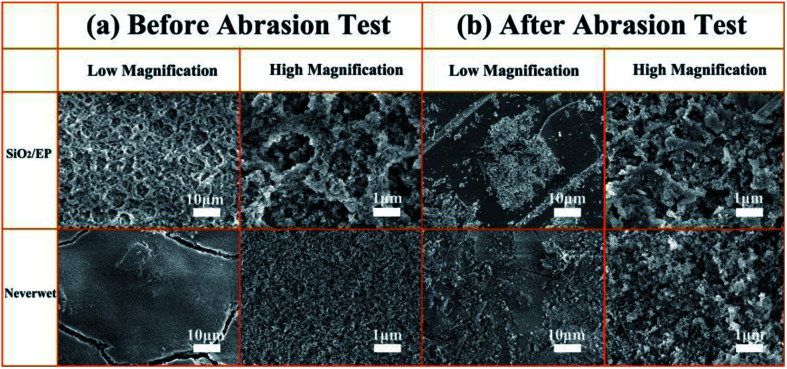
SEM images of SiO_2_/epoxy resin coatings and Never Wet before and after abrasion tests for 4000 cm.

To further quantify the abrasion-resistance of these materials, water contact angle, sliding angle and retention ratio were plotted as a function of sandpaper abrasion distance on the SiO_2_/epoxy resin coating and Never Wet, respectively, as shown in Fig. 7. It is clear that after a 4000 cm abrasion distance, the retention ratio was about 78% for the obtained SiO_2_/epoxy resin coatings, whilst, the retention ratio was about 58% for the Never Wet coating, which indicates that the SiO_2_/epoxy resin coating has better abrasion resistance than the commercial coating (Never Wet). Their surface morphologies did not significantly change before and after abrasion for 4000 cm of distance as shown in Fig. 8, and the surface always maintained its rough structures, which is necessary for superhydrophobicity. The elemental mapping of C, N, O and Si before and after the abrasion test are shown in Fig. S6 in the ESI.† It is clear that all the elements are distributed on the coating surface uniformly after abrasion, suggesting the excellent abrasion resistance.

### 3.3 Oil and water separation

**Fig. 9:**
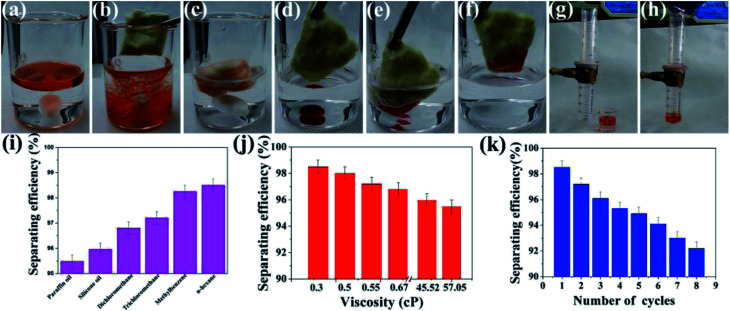
(a–h) Oil/water separation by coated cotton and window screening mesh. (i) Separation efficiency for different oil and solvents. (j) Relationship of separation efficiency and reuse numbers. (k) Relationship between viscosity and separation efficiency.

The coating can be fabricated on a porous window screening mesh or cotton *via* a dip-coating method and the superhydrophobicity as well as superoleophilicity of the coating make it a good candidate for oil/water separation. As shown in Fig. 9(a–c) and Video S1 in the ESI,† if stirred with an oil/water mixture solution at a high speed for a few seconds, the cotton could totally separate the water and oil. The obtained coating can also be used to separate heavy oil, and the oil could be absorbed immediately into the cotton on contact (Fig. 9(d–f) and Video S2 in the ESI†). In addition, the robust coating can be fabricated on a window screening mesh and be used to separate oil/water mixture solutions, as shown in Fig. 9(g and h) and Video S3 in the ESI.† Note that the coating demonstrates stable superhydrophobicity no matter how the window screening mesh was abraded, as shown in Fig. S7.†Fig. 9(i) shows the different separating efficiencies of the superhydrophobic window screening mesh to various solvents and oils. For the oils and solvents with different densities and viscosities, such as paraffin oil or *n*-hexane, the separating efficiency was above 95%. It is clear that the separating efficiency increases with lower viscosities, as shown in Fig. 9(j). It is believed that the oils or organic solvents with high viscosity (*e.g.*, paraffin oil) tended to block the pores of the coating, resulting in a lower separation efficiency. After being rinsed thoroughly with alcohol and dried, the as-prepared window screening mesh could be reused for oil/water separation after many cycles and maintained its separating efficiency above 90% (Fig. 9(k)).

## 4. Conclusion

In conclusion, we have demonstrated a facile and low-cost method to fabricate superhydrophobic coatings by anchoring functionalized SiO_2_ nanoparticles to epoxy resin coatings based on co-curing and thus avoiding phase separation between inorganic nanoparticles and the organic epoxy resin. The fluorine-free coatings have strong adhesion to the substrates and also demonstrate excellent mechanical abrasion resistance and anti-corrosion properties. The coatings can also be fabricated on porous substrates and be used to separate oil and water mixture solutions efficiently. It is expected that the super-robust and strong coatings with durable superhydrophobicity will have a wide range of applications for outdoor use.

## Conflicts of interest

There are no conflicts of interest to declare.

## Supplementary Material

RA-009-C8RA10046B-s001

RA-009-C8RA10046B-s002

RA-009-C8RA10046B-s003

RA-009-C8RA10046B-s004
