# Supplementary material for: Reactive silica nanoparticles turn epoxy coating from hydrophilic to super-robust superhydrophobic
Source: RSC Adv. 2019 Apr 24;9(22):12547–54. doi: 10.1039/c8ra10046b (PMC9063669; doi:10.1039/c8ra10046b)
Supplement: RA-009-C8RA10046B-s001 [file RA-009-C8RA10046B-s001.pdf]

Electronic Supplementary Material (ESI) for RSC Advances.
This journal is © The Royal Society of Chemistry 2019

Supporting information for

Reactive silica nanoparticles turn epoxy coating from hydrophilicity to super-robust superhydrophobicity

Danfeng Zhi[1#], Huanhuan Wang[1#], Dong Jiang[2], Ivan P. Parkin[3], Xia Zhang[1*]

[1] Engineering Research Center for Nanomaterials, Henan University, Kaifeng 475004, People's Republic of China, Kaifeng 475004, PR China.

[2]State Key Laboratory of Solid Lubrication, Lanzhou Institute of Chemical Physics, Chinese Academy of Sciences, Lanzhou 730000, PR China

[3]Department of Chemistry, University College London, 20 Gordon Street, London, WC1H 0AJ, UK.

# Equal contribution.

* Corresponding author. Email: xia.zhang@ucl.ac.uk

S1. Formation and Characterization of surface functional silica nanoparticles

First, sodium metasilicate is hydrolyzed to form the silicic acid under the existence of HCl. Condensation polymerization happens by hydration among the hydroxyls of silicic acids at three dimensions and the Si and O are bonded to each other to form nanosilica clusters. Large numbers of hydroxyls are left on the surface of the nanoclusters. In the meantime, KH560 is hydrolyzed to produce trimethylsilyl. As soon as the nanoclusters are formed, the trimethylsilyl reacts rapidly with the hydroxyl of nanosilica to form the modification layer on the surface of the clusters. Here, the trimethylsilyls substitute a majority of active groups of silica, which prevents silica from continuously growing up or agglomerating. Thus, the surface of hydrophilic silica nanoparticles was endowed reactive groups and could further cure along with the epoxy resin. From the XRD spectrum (Fig. S1a), it can be seen that there is a broad peak which indicates the obtained silica is amorphous. Fig. S1b shows the SEM image of the silica nanoparticles and it can be seen that the size of silica nanoparticles are several tens of nanometers and aggregate with each other. XPS analysis shows that the coating mainly consists of C, O, Si and do not contain any other elements. The white powder has a

good dispersion in organic solution and can form a clear solution when dispersed into ethanol (Fig. S1d inset).

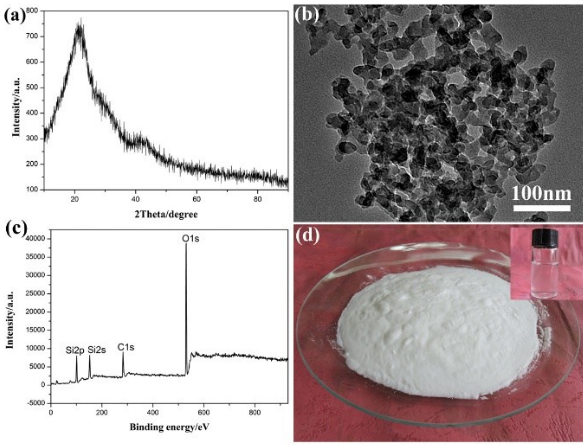

Fig. S1. (a) The XRD spectrum and (b) TEM images of the silica particles; (c)XPS analysis; (d) Photograph of the obtained silica powder and the powder dispersed into ethanol solution (inset).

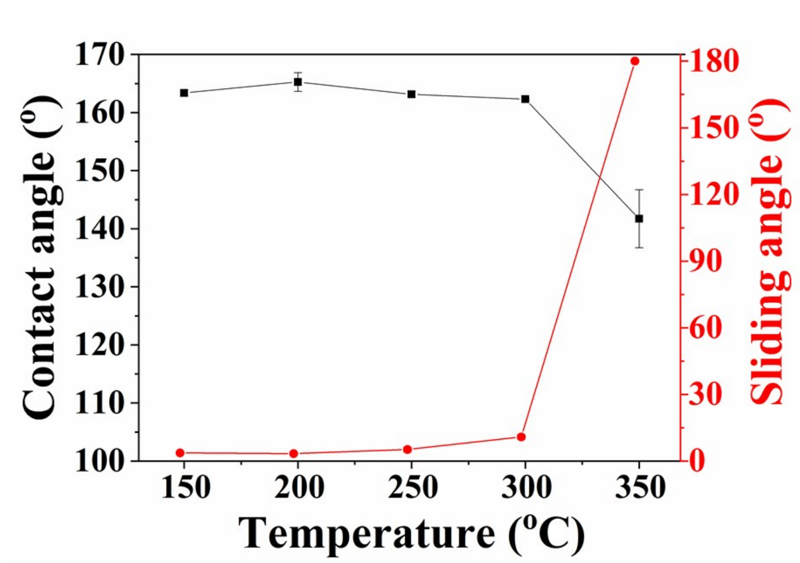

Fig. S2. The relationship between water contact angle, sliding angle and the curing temperature.

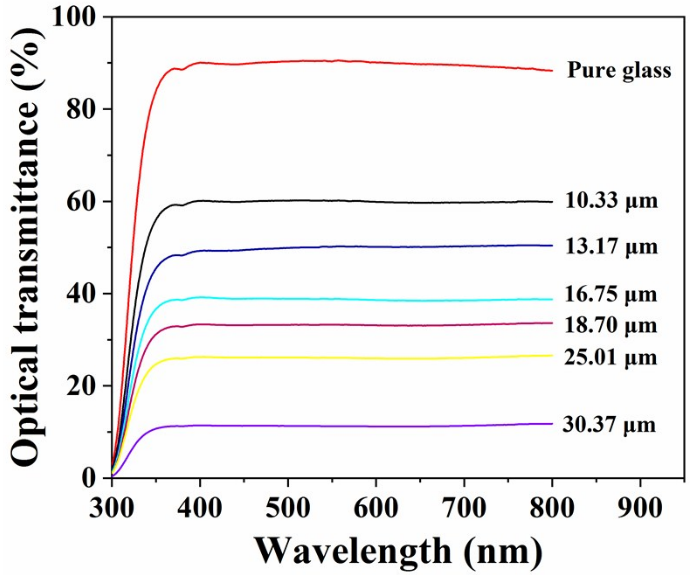

Fig. S3. The optical transmittance of the coating with different thickness.

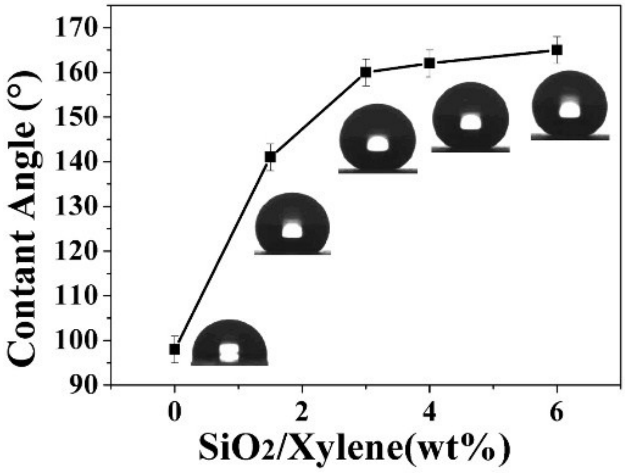

Fig. S4. The relationship between water contact angle and the mass fraction of SiO_2 nanoparticles in the solution.

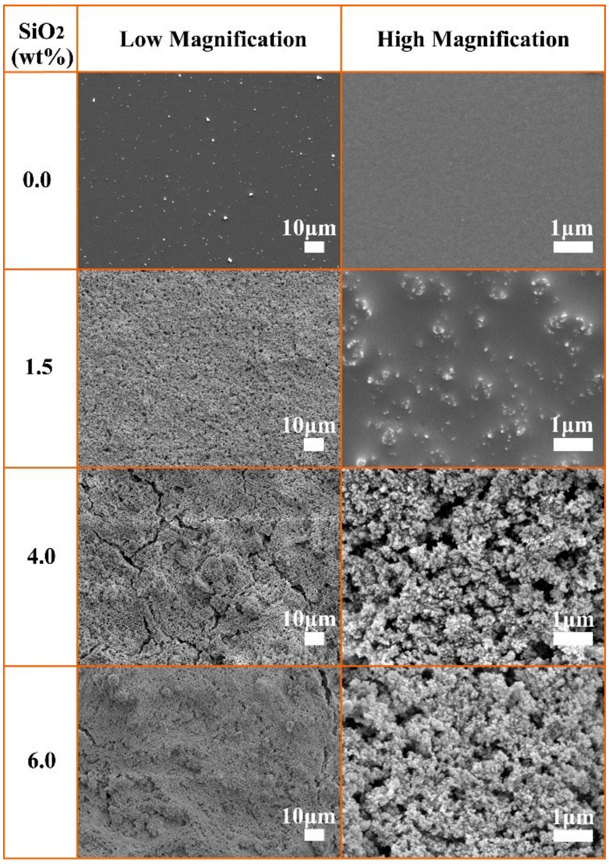

Fig. S5. SEM images of coating surface with different SiO_2 addition.

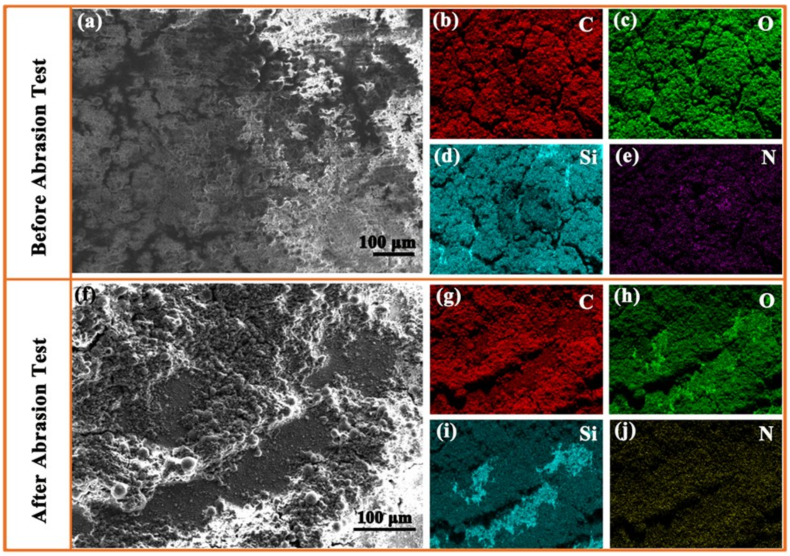

Fig. S6. The mapping of the coating before and after abrasion test.

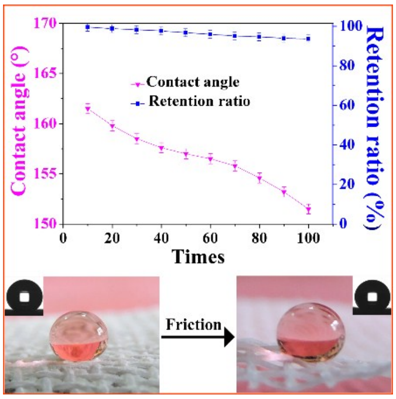

Fig. S7. Plot of water contact angle and retention ratio as a function of sandpaper abrasion times on SiO_2/epoxy network coatings. Photograph of a water droplet sitting on the coating before and after abrasion with water contact angle about 158° (before friction) and 151° (after friction).
